# EXPLORATION OF FALL RISK OF PARTICIPANTS ACROSS EVIDENCE-BASED FALL PREVENTION PROGRAMS

**DOI:** 10.1093/geroni/igad104.3327

**Published:** 2023-12-21

**Authors:** Rita Wong, Cathy Elrod, Sara Pappa, Uma Kelekar, Patricia Heyn

**Affiliations:** Marymount University, Arlington, Virginia, United States; Marymount University, Arlington, Virginia, United States; Marymount University, Arlington, Virginia, United States; Marymount University, Arlington, Virginia, United States; Marymount University, Arlington, Virginia, United States

## Abstract

The National Council on Aging houses the Administration for Community Living’s (ACL) national database on evidence-based, community-delivered fall prevention programs (EBFPP). Each year, cooperative agreement grants are awarded competitively to groups demonstrating readiness to deliver EBFPP in their communities. Participants in these programs completed a standardized pre-participation survey documenting demographic and self-reported fall risk factors that were entered into the national EBFPP database. Marymount University received permission to analyze the survey data entered into the ACL’s National Falls Prevention Database between July 2016 and June 2022. An exploratory analysis was conducted of 105,323 older adults who participated in one of eight ACL-approved EBFPP programs. EBFPPs vary in their targeted fall risk level, tailoring intervention components (health education, self-management training, physical exercise) and adjusting intensity and focus to match the risk level. It is unknown if people join the program that best fits their risk level. This study evaluates and compares participant demographic characteristics and fall risk across EBFPPs. We created an index to categorize risk levels, anchored in the CDC’s STEADI toolkit for fall risk, finding that 29% were high risk, 51% moderate risk, and 19% low risk. The factors used to establish risk level included 1) falling in the last 3 months, 2) referral from a healthcare professional, 3) fear of falling, 4) self-reported health (not excellent or very good), 5) depression, and 6) age 85 or older. Exploring participant characteristics by risk level and choice of EBFPP adds unique information to guide future EBFPP recruitment strategies.

